# Experiences of friendship among autistic adults: a scoping review

**DOI:** 10.3389/fpsyt.2025.1523506

**Published:** 2025-04-14

**Authors:** Tian Wu, Duu-chiang Wang

**Affiliations:** ^1^ Department of Rehabilitation Sciences, Nanjing Normal University of Special Education, Nanjing, China; ^2^ Department of Social Work, Tunghai University, Taichung, Taiwan

**Keywords:** adult, autism spectrum disorder, friendship, experience, coping review

## Abstract

**Aims:**

In this review, we comprehensively mapped the literature on the experiences of friendship among autistic adults

**Data sources:**

A scoping review was conducted following databases from the earliest records to December 2023 in four electronic databases (PubMed, ERIC, Web of Science and EBSCO (Psychology and Behavioral Sciences Collection, APA PsycArticles, APA PsyInfo, and Open Dissertations) to (a) identify the quantity, breadth, and methodological characteristics of the literature, (b) summarize and synthesize key research findings, and (c) explore knowledge gaps to guide future research.

**Results:**

A total of 22 empirical studies were included. The results indicated that the most frequently studied components were friendship status; friendship practice; meaning of friendships; relationships between friendship and other factors.

**Conclusion:**

Future studies should incorporate the voice of autistic adults and focus on the dynamics and contexts of friendship experiences.

## Introduction

Friendship is an essential social relationship formed over the life span of almost all individuals. Friendship, which is based on interpersonal interactions, encompasses unique behaviors accompanied by a range of emotions, hopes, regrets, and wishes (1), and it transcends the boundaries of age, gender, and settings (2). From the perspective of social psychology, friendship is regarded as a specific form of a dyadic peer relationship, which is dynamic, stable, voluntary, and reciprocal in nature (3). According to Hall (4), symmetrical reciprocity, agency, enjoyment, instrumental aid, similarity, and communion are the six factors of expectation that constitute the optimal standards of friendship. As a dynamic relationship that develops within a specific period in a given environment, friendship involves a degree of mutual affection and companionship (5–7). Friendship experiences affect not only individuals’ health, emotional well-being, social interactions, and cognitive functioning but also their families, school performance, and entire neighborhoods (4, 8). Therefore, the complexity of friendship experiences is reflected not only in the static structural network of friendship but also in its dynamic formation process (9).

In the most recent revision of the Diagnostic and Statistical Manual of Mental Disorders (Fifth Edition) (10), the umbrella term “autism spectrum disorder (ASD)” was introduced, and characterized by two domains, including differing social and communicative skills, and restrictive behaviors, interests, and activities. It has been a consistent finding that these core characteristics of autism will affect the formation, maintenance and outcomes of social connection, such as friendship and peer relationship, throughout the life span (11, 12). Typically, for young individuals with disabilities, friendships and personal relationships are as an essential component for achieving a successful transition to college and career life (13, 14). Autistic individuals may struggle with making social relationship at all ages. Unlike childhood and adolescence, other symptoms may be improved yet social impairment will be more prominent in adulthood. Autistic individuals live the majority of their years during adulthood in the complex context (the complexity and diversity of interpersonal relationships), especially when leaving school and transitioning to workplace (15). From a life-span theory, the role of friendship varies at different stages of life, such as involved in subjective well-being in late life while focused on social cognition in childhood (1, 16). The studies focused on the experiences of friendship for autistic individuals reported the various challenges and concerns at different ages, especially the increased wanting to fit in and have friends in autistic adolescents (17, 18). There was also some evidence that greater quantity and quality of friendships were associated with decreased loneliness among autistic adolescents and adults, especially the number of friends provided protective role in predicting self-esteem, depression, and anxiety (19, 20).

A number of systematic reviews into this topic have been conducted over the past ten years. Much of the research examined the friendship or peer relationships between children and adolescents, (21–24), or across the lifespan (11), and describe or explain friendship through a certain perspective, including the internal structure and quality of friendship, the static status and dynamic process of friendship. Petrina et al. (24) reviewed 24 studies of the nature of friendships among autistic children. They discovered major differences in the manifestation of friendships between autistic children and their neurotypical (NT) peers, including friendship characteristics, definitions of friendship, friendship quality, reciprocity of friendship, and friendship satisfaction. In a meta-analysis, Mendelson et al. (23) reviewed 18 papers to explore the descriptive friendship literature among school-age [school age (6 –12) and adolescence (13–17)] boys with ASD. In total, the study included 1,768 participants, 85.46% of which were males with a mean age of 9 years and 7 months. They discovered that autistic boys had fewer and lower-quality friendships than their NT peers. The core driver of these differences came from social information processing speed (SIPS), which refers to the capacity to comprehend and respond appropriately during social interactions. Therefore, they developed a process-based model of friendships which building on the tenets of Hartup and Stevens (25) model, for better understanding the processes of friendship among autistic boys, and suggested that school-age boys with ASD struggle to form deeper individual and reciprocal friendships with their peers. In a systematic review, Brady et al. (21) examined the interventions used to teach friendship-related social skills to autistic children and adolescents Given that these studies only focused on children and adolescents, further research is required to investigate the experiences of friendships among different age groups on the autism spectrum, especially among adults. Collectively, the aforementioned reviews included both qualitative and quantitative studies (11, 22). The methods used in the studies were mainly quantitative, and some studies combined quantitative and qualitative methods.

Unlike a systematic review, the aim of a scoping review is to provide an overview on broader topics beyond those related to the effectiveness of an intervention and bringing together literature with emerging evidence (26–28). Through a systematic approach, scoping reviews examine the extent or nature of evidence on a specific topic, summarize findings, and identify gaps in the literature, thereby facilitating the mapping of evidence, theories, concepts, and sources to aid in the planning of future research (29, 30).

Despite increasing knowledge on the topic, to the best of our knowledge, no systematic international review has examined the experiences of friendships among autistic adults. This review specifically focused on the experience of autistic adults and included qualitative and quantitative studies, which are underrepresented in previous reviews. Given that scoping reviews are used to comprehensively map existing research, we conducted this scoping review to achieve the following goals:

- Identify the quantity, breadth, and methodological characteristics of the literature on the experiences of friendships among autistic adults,
- Summarize and synthesize key research findings, particularly regarding the characteristics of friendships from the perspective of individuals on the autism spectrum, and
- Explore the gaps in the literature to guide future research.

## Methods

This scoping review was conducted in accordance with the guidance framework of Arksey and O’Malley (29) and the Preferred Reporting Items for Systematic Reviews and Meta-Analyses (PRISMA) framework and the extension for scoping reviews (31–33). Scoping reviews are typically conducted in five stages: identifying the research question; identifying relevant studies; selecting studies; charting the data; and collating, summarizing, and reporting the results (29). In the following, we describe each of these stages in detail.

### Stage 1: identifying the research question

Our research question was as follows: What information does the literature provide regarding the perceptions, experiences, and nature of friendships among autistic adults?

### Stage 2: identifying relevant studies

In this review, we conducted a systematic search to identify studies relevant to our research question using the following databases from the earliest records (October 2003) to December 2023: PubMed, ERIC, Web of Science and EBSCO (Psychology and Behavioral Sciences Collection, APA PsycArticles, APA PsyInfo, and Open Dissertations). One set of search terms describing participants (“adult” OR “adulthood” * AND “autism” OR “Asperger” OR “autistic” or “ASD”*) was combined with a second set of keywords describing friendship (“friendship” OR friend “OR “make friends”*) in all possible permutations. In addition, to avoid the risk of omitting relevant studies, a hand-search of the reference lists of all included studies and Google Scholar (search term was “friendships among autistic adults” and sort by relevance) was also conducted.

### Stage 3: selecting studies

This scoping review was conducted using the systematic review method, and a *post hoc* study, which refers to an analysis conducted to explore the themes not based on pre-specified hypotheses before the study began, was also performed on the basis of the researchers’ increased familiarity with the literature on the experiences of friendships among autistic adults.

Studies were included if they (a) focused on the experiences of friendship among autistic adults, (b) employed participants age > 18 who had an ASD diagnosis. (c) provided empirical data and using qualitative or quantitative methods or mixed methods.

And articles were excluded if they (a) focused on interventions or therapy rather than the nature of friendships (e.g. (34, 35), or (b) examined other related concept (e.g. loneliness (36), social relationship (37) and the research topic did not involve friendships, or (c) examined all of the participants aged<18 (e.g. 38–40), or (d) all of the participants were non-clinical individuals [e.g. (41, 42)] or other disorder.

After the removal of duplicates, the titles and abstracts of all retrieved articles were screened. Articles were selected or excluded in accordance with the inclusion and exclusion criteria (interrater reliability 96%). Finally, the full texts of all articles were examined to reach a final decision regarding their inclusion in this scoping review (interrater reliability 94%). Two authors conducted the process independently and then checked agreement. The reliability of the searching was determined by comparing the number of articles identified by the two reviewers. Three articles with different opinions were discussed in more depth between the two reviewers and resolved through reconsidering inclusion to reach a consensus. 22 articles deemed eligible for inclusion were reviewed and agreed upon by all authors.

### Stage 4: charting the data

In line with the research question, the descriptive characteristics of the selected articles were extracted by the reviewers in an iterative manner, including the author name(s), year of publication, study location, study population, and study aim, methodology, and outcomes. The main findings related to the experiences of friendships among autistic adults were charted. The key items of information were collated by the reviewers in a customized data extraction sheet.

Two independent coders extracted and coded data from 22 papers using a the thematic approach developed by Clarke and Braun (43) and refined by Kiger and Varpio (44). First, all 22 papers were encoded by two independent coders (first and second author) to search on demographic variables (the range of TD and ASD participant age; proportion of TD and ASD samples that were male). Second, two authors read each study multiple times to identify codes, which were guided by the research question, and then these codes were subsequently utilized to construct key concepts and themes, including friendship status; friendship practice; the meaning of friendships and the relationships between friendship and other factors.

The content of the coding mainly included self-reported friendship quality (companionship, security, closeness, providing help or support), and parent-reported friendship quantity (number and duration of friends and reciprocal friendship). Various components of friendship experiences were identified and grouped under overarching themes. The key themes were organized into an inductive conceptual framework on the basis of discussions of synthesized results between all reviewers.

### Stage 5: collating, summarizing, and reporting the results

To develop a framework for collating and summarizing the results, certain aspects of the literature were prioritized in both quantitative and qualitative analyses. The results regarding the author’ name(s), publication year, study location, study population, and study aim, methodology, and outcomes were summarized in a chart format (**Table 1**). Because of the variations observed in the outcomes and main findings between the articles, a narrative synthesis format was selected to discuss the results.

**Table 1:** Data charting.

Authors,year, country	Aims	Participant characteristics	Method	Main Findings
Baron-Cohen and Wheelwright (2003) (45), UK	To report a new self-report questionnaire, the FQ, for use with adults. To test the theory that autism is an extreme form of the male brain.	Study 1: 76 adults (aged 18.0–58.7 ;27 males, 49 females) from a general population; Study2: 68 adults and adolescents with AS/HFA (aged 14.0–63.9 years; 51 males, 17 females).	Questionnaire: FQ	▪ The adults with AS or HFA scored significantly lower on the FQ than both the male and female from controls. ▪ The FQ thus reveals both a sex difference in the style of friendship in the general population, and provides support for the extreme male brain theory of autism.
Chan et al. (2022) (46), USA	To describe the range of social participation experiences of autistic adults from the individual’s perspective.	40 autistic adults (aged 24–62; 27 males, 13 females, IQ>70).	Qualitative data from semi-structured interviews.	Five main contexts where social participation occurred: (1) Vocational contexts, (2) Neighborhoods, (3) Common interest groups, (4) Support services and inclusive environments, and (5) Online networks and apps.
Crompton et al. (2020) (47), UK	To explore and contrast autistic experiences of spending social time with neurotypical and autistic friends and family	12 autistic adults (aged 21–51; 2 males, 10 females)	Qualitative data from semi-structured interviews.	Three themes were identified: cross-neurotype understanding, minority status and belonging. Revealed the need for autistic-led social opportunities and indicate benefits of informal peer support for autistic adults.
DaWalt et al. (2019) (19), USA	To examine the experiences of friendships and social participation of individuals with fragile X syndrome and autistic disorder during adolescence and adulthood.	81 adolescents and adults with FXS(37 Teens, 44 adults, aged 17-35); and 226 adolescents and adults with AD and ID (106 teens, 120 adults; aged 14-45).	Longitudinal survey data from National Survey of Families and Households/ ADI-R	▪ Individuals with fragile X had more friendships and a less negative social impact on the family than individuals with autism. ▪ Adolescents spent less time with friends and neighbors, and more time in exercising, than did adults.
de Carvalho (48), Portugal	To employ a new Portuguese version of the FQ together with other instruments (AQ and EQ).	531 participants (n=33 diagnosed with ASD, n= 498 from general population); (aged 18-74;134 males, 397 females)	Questionnaire and Factor analyses for the FQ, together with the AQ, the EQ, and the SQ.	▪ The FQ was most strongly related to the EQ. ▪ All the instruments can discriminate between a normal and an ASD population, the most important source of discrimination being the AQ, followed by the FQ.
Finke (2022) (49), USA	To explore the preferred behaviors of autistic and non-autistic young adults with respect to making and keeping friendships.	102 autistic (aged 18-24; 73 males, 29 females) and 107 non-autistic young adults (aged 18-24; 77 males, 30 females).	Questionnaire, comprised of selected questions from the FQ	Results identified differences in the preferred friendship practices between the autistic and non-autistic young adults, e.g. (1) autistic young adults would rather talk on the phone with a friend to make arrangements\meet up with a friend for a specific activity, compared to people without an ASD diagnosis prefer to talk on the phone with a friend\meet up with a friend just to chat. (2) autistic individuals more likely to report their friends value them as someone to have fun with, compared to people without an ASD diagnosis reported their friends value them as someone to support them.
Finke et al. (2019) (50), USA	To identify the similarities and differences in the broad perspectives and friendship practices of young adults with and without autism	126 young adults with autism (aged18-24; 89 males, 37 females) and 125 young adults without autism (aged 18-24; 35 males, 90 females)	Questionnaire: FQ	Young adults with autism would like to have more friends. Individuals with autism at the group level, expressed different preferences for their friendships than their non-autistic peers. Physical closeness or physical distance is the defining factor in friendship preferences that most define the perspectives of young adults with autism.
Forster and Pearson (2019) (51), UK	To explore social relationships and understanding of the concept of mate crime in autistic adults.	5 autistic adults (aged 22-25; 3 males, 2 females)	Semi-structured interviews and IPA	Three superordinate themes: (1). learning the formula (2). Socialising… (3). Taking Advantage of You
Friedman et al. (2019) (52), USA	To examine conversational language and its impact on vocational independence and friendship status in adults with ASD	84 adults with ASD (aged 18–53; 62 males, 22 females) and their parents.	interview with an examiner as language samples, questionnaires: ADI-R\IQ.	▪ Conversational language abilities in adulthood predicted functional outcomes. ▪ Specifically, Vocabulary diversity was predictive for both vocational independence and friendship outcomes.
Gallup and Serianni (2017) (53), USA	To explore emotional expression and awareness in the context of a virtual environment specific to young adults with ASD.	5 young adults with ASD (aged 19-24; 3 males, 2 females)	personal interviews, scheduled observation and document analysis	Prominent theme: Recognizing and reciprocating emotions and emotional awareness: (1)Seeking Social Interaction and Defining; (2)Emotional awareness (3)Roles in life, Increased socialization and Friendships; (4) Skills Learned
Johnson (2014) (54), Canada	To examine the experiences of adults with autism as they attempt to develop and maintain friendships and romantic relationships.	26 individuals (aged 16+ with a mean age of 31, 12 males, 14 females)	Mixed survey contained multiple choice questions and open-ended questions.	The participants reported varying but generally high levels of social and romantic interest. What that social connection might look like could vary greatly both within this group and as compared to “neurotypical” persons. The apparent disparity observed between desired time spent with friends versus the actual time spent with friends.
Mazurek (2014) (20), USA	To examined the relations among loneliness, friendship, and emotional functioning in adults with ASD.	108 adults with ASD (aged 18-62; 57males, 51 females)	Questionnaires: AQ-Short\ULS-8\ URCS\ SWLS\ RSE\PHQ	▪ loneliness was associated with increased depression and anxiety and decreased life satisfaction and self-esteem, even after controlling for symptoms of autism spectrum disorders. ▪ Greater quantity and quality of friendships were associated with decreased loneliness among adults with ASD. ▪ Number of friends provided unique independent effects in predicting self-esteem, depression, and anxiety above and beyond the effects of loneliness.
Pearson et al. (2022) (55), UK	To explore experiences of interpersonal victimization among autistic adults by familiar others from their own perspective.	64 autistic adults (aged 14–52;13 males, 27 females, 2 nonbinary people, and 1 genderqueer person)	qualitative online study	Two key themes were identified, (1) cycles of victimization’’ highlighted the occurrence of polyvictimization in the sample. (2) perceptions of victimization focused on how these experiences were related to difficulties with trust (of both self and others), the recognition of victimization, and heightened compliance.
Płatos and Pisula (2021) (12), Poland	To examine friendship understanding in adolescents and adults	76 adolescents and young adults on the autism spectrum (aged 14-37; 44 males, 32 females) and 76 typically developing individuals (aged 14–34; 41 males, 35 females)	Qualitative data from open-ended questions	▪ Autistic people referred to intimacy and unconditional responsiveness less often and also provided less complex definitions of a ‘friend’ than their typically developing peers. ▪ There were some distinct profiles of friendship understanding in gender and cognitive-developmental
Rossetti (2014) (56), USA	To explore the connections and dynamics of friendships among three groups of secondary school-aged young adults.	Three groups (n=3,2,2) of young adults; each group includes an individual with autism or severe disability and nondisabled	Qualitative data from naturalistic observations and semi-structured interviews	The thematic findings included: (a) excitement and motivation, (b) shared humor, (c) normalized supports, (d) mutual benefits, and (e) differing conceptions of friendships.
Rossetti (2011) (57), USA	To explores the contexts and dynamics of friendships among three groups of young adults	Three groups (n=3,2,2) of young adults; each group includes an individual with autism or severe disability and nondisabled (2 autism, 4 nondisabled, 1 Menkes syndrome).	Qualitative data from naturalistic observations and semi-structured interviews	Three key components of how these friendships were enacted: (a) the connections of friendship, (b) the difficulties in maintaining friends, (c) the examples of friendship work.
Rossetti (2012) (58), USA	To explores the contexts and dynamics of friendships among three groups of young adults	Each group included an individual with autism or severe disability and high school students without disabilities.	Qualitative data from naturalistic observations and semi-structured interviews	Key themes: (a) Educator influence on student friendships that decreased interactions included two categories of factors: missed opportunities and type of academic participation; (b) Educator influence that increased interactions included specifically four strategies: (1) build bridges (2) adult as mentor,(3) student as mentor and (4) fade back, and classmates fill the spaces.
Sedgewick et al. (2019) (59), UK	To examine the nature of the friendships, relationships, and conflict within the relationships of autistic and neurotypical adult women	38 women (aged 20 -40, 19 autistic, 19 neurotypical women)	mixed methods: questionnaires (URCS, TASIT), and semi-structured interviews.	▪ The social relationships and experiences of autistic women were much like those of neurotypical women. ▪ Autistic women had greater difficulty with social inference skills, and reported experiencing more negative social situations.
Sedgewick et al. (2019) (59), UK	To examine gender differences in FQ between the autistic and non-autistic adults; and comparing FQ results to URCS	931 participants (532 autistic: aged 18-71; 72 males, 317 females, 143 NBT); (391 non-autistic: aged 18-81; 54 males, 327 females, 18 NBT)	Questionnaires: AQ\FQ\URCS	▪ Autistic people score lower on the FQ. ▪ There were gender differences among the autistic population. ▪ Autistic people scored on the URCS more highly than non-autistic adults did.
Sosnowy et al. (2019) (60), USA	To describe the perspectives of young adults on the autism spectrum about how they seek and make friends in diverse ways that develop satisfying friendships.	20 young adults on the autism spectrum (aged 18-29; 11 males, 7 females, 2 NBT) and their parents	Semi-structured interviews, and grounded theory	Themes: (a) Navigating social norms as persistent efforts to make friends; (b) Finding friends who accept their differences; (c) Shared interests where the autism was not necessarily a central concern.
Sundberg (2018) (61), Hungary	To investigate the possible links between online gaming, loneliness and friendships	151 participants: 85 adolescents and adults with ASD (aged 14-60; 49 males, 36 females) and 66 adolescents and adults (aged 15-69; 34males, 32 females)	Questionnaires: MOGQ\ ULS-8\ URCS	▪ Within the ASD sample, persons who play online games have more friends than those who do not. ▪ Motives to play online games differed between the ASD sample and the control group. ▪ Friendship quality and having a best or close friend were not linked with online gaming.
Worrell (2017) (62), USA	To discover constructions between friendship and victimization among emerging outcomes with HFASD and to identify the functional role of friendship plays in the lives of emerging outcomes with autism.	7 participants (aged 19-32, 4 males, 3 females)	Semi-structured interviews and IPA	seven themes included: differences between HFASD individuals and neurotypical individuals, family members identified as their best friends, loners, challenges in friendships, bullies and the primary age/grade that childhood friendship struggles are the most prevalent.

AQ, Autism Quotient; EQ, Empathy Quotient; SQ, Systematization Quotient; URCS, Unidimensional Relationship Closeness Scale; TASIT, The Awareness of Social Inference Test; Asperger Syndrome or high-functioning autism; FXS, Fragile X Syndrome; ID, Intellectual Disabilities; AD, Autistic Disorder; ADI-R, Autism Diagnostic Interview – Revised; IPA, Interpretive Phenomenological Analysis; ULS-8, 8-item version of the UCLA Loneliness Scale; SWLS, Satisfaction with Life Scale; RSE, Rosenberg Self-Esteem Scale; PHQ, Patient Health Questionnaire; NBT, Non-Binary Transgender; MOGQ, Motives for Online Gaming Questionnaire.

The two authors independently read each study multiple times and extracted the key sentences to form codes, following which generating a set of statements to identify key concepts and themes. Emerging concepts and themes were discussed regularly throughout the process due to the potential researcher bias. The themes identified were compared across studies to explore deeper and latent relationships from concepts and themes between studies; these phases were discussed between the two authors until a consensus was reached (interrater reliability of 97%).

## Results

### Search results

**Figure 1:**
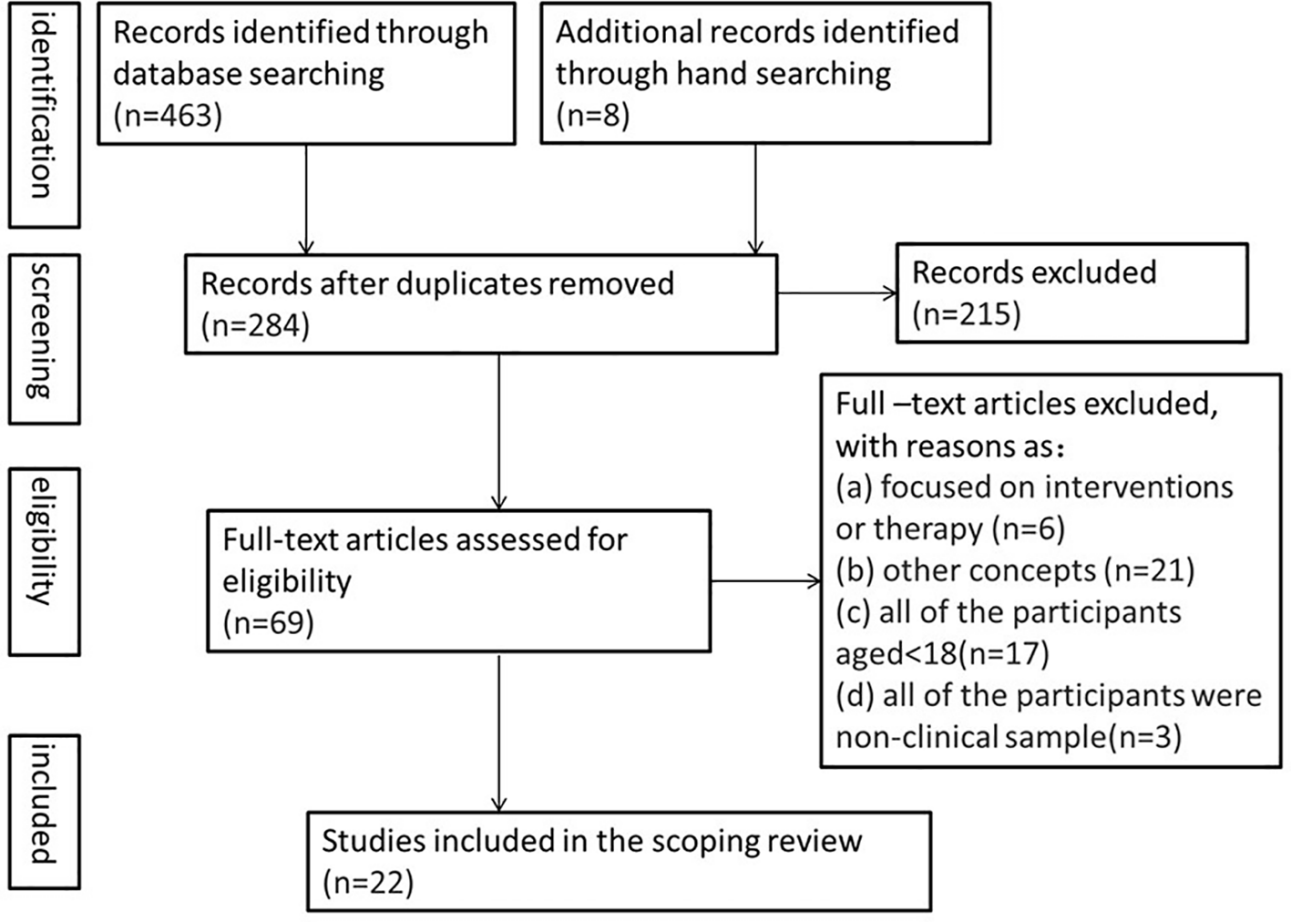
PRISMA flowchart of the study selection process.

Initially, a total of 463 articles were identified through electronic database searching and manual searching. After the removal of duplicates, 284 articles remained and the titles and abstracts of them were screened. 215 articles were excluded in accordance with the inclusion criteria (interrater reliability 96%). Finally, the full texts of the remaining 69 articles were examined to reach a final decision. 47 articles were excluded at full-text review due to the excluded standard (interrater reliability 94%). **Figure 1** shows the PRISMA flowchart of the study selection process.

### Study characteristics

**Table 1** presented the general characteristics of these studies. Most of the studies were conducted in the USA (54.6%; *n*=12) and the UK (27.4%; *n*=6), followed by Canada (4.5%; *n*=1), Hungary (4.5%; *n*=1), Poland (4.5%; *n*=1), and Portugal (4.5%; *n* = 1) (see **Table 1**). The majority of studies were published between 2011 and 2023 (95.5%; *n*=21), reflecting the emerging research status of this field.

Nearly half of the studies employed qualitative methods (45.5%; *n*=10), and nine papers used quantitative methods with the remaining three studies adopting a mixed-method approach. The scoping review identified three main measures with potential use for friendships among autistic adults across the quantitative (40.9%; *n*=9) and mixed research (4.5%; *n*=1) studies, including two standardized self-report questionnaires (Friendship Questionnaire, FQ) (45) and Unidimensional Relationship Closeness Scale, URCS) and one parent-report interview (Autism Diagnostic Interview-revised: a revised version of a diagnostic ADI-R) (63). Among them, eight studies were self-reported by autistic adults using FQ (18.2%; *n*=4), or URCS (13.6%; *n*=3) or both (4.5%; *n*=1), while two studies were reported by parents using ADI-R (9.1%; *n*=2).

Most of the qualitative studies used only semi-structured interviews (27.3%; *n*=6), or both interviews and observations (18.2%; *n*=4). Of these studies, three involved phenomenological analysis (IPA), and one relied on grounded theory for analysis. **Table 1** listed the characteristics of the methodologies used in all the included studies.

### Participant characteristics

A total of 2102 participants were included across the 22 studies, among whom 768 were males, 1169 were females and 164 were gender non-binary, one was genderqueer. The age of participants spanned 14 to 81 years. One of the studies used a female-only sample. The diagnosis was established by psychiatrists or confirmed through psychological reports (or parents) previously. Five of the studies reported the participants’ IQ in the normal range. Five of studies included autistic-related disorders, including, pervasive developmental disorder (*n*=42) and ASD (*n*=253).

And nine of the studies reported the participants’ ethnic background. The primary race was white (84.8%; *n*=446). Two of the studies included informants (parents) in addition to the autistic adults as participants and one paper investigated only mothers. Of the 7 comparative studies, 6 compared autistic adults to neurotypical population, and 1 compared autistic adults to fragile X syndrome. Two of the studies reported friendship relations that occurred within online settings.

### Main findings

Experiences of friendships are diverse and complex among autistic adults. The nature of friendships and related factors were identified and coded into initial themes. Through discussions and collaborations, these themes were organized into an inductive conceptual framework describing five central components of friendships among autistic adults:

- Friendship Status
- Friendship Practice
- Meaning of Friendships
- Relationships between friendship and other factors

### Friendship status

Five quantitative and two mix-method studies provided data on the status and closeness of friendship in autistic adults, including four comparative and three non-comparative studies. With respect to tools, URCS (*n*=4) and ADI-R (*n*=2) were used to measure it and one mixed-method study used the self-compiled questionnaire. Among them, five studies were self-reported by autistic adults using URCS (*n*=4) and self-compiled questionnaire (*n*=1), while two studies were reported by parents of autistic adults using ADI-R.

A total of seven studies utilized self-reported data from autistic adults, comprising six comparative studies and one non-comparative study. These studies included a combined sample of 1,114 autistic and 1,241 non-autistic participants. Among these, 782 autistic and 924 non-autistic participants from four studies reported scores on the FQ scale. Autistic participants scored M = 61.82 (SD = 19.30), while non-autistic participants scored M = 76.69 (SD = 14.80). Additionally, 212 autistic and 85 non-autistic participants from four studies provided scores on the URCS scale. Autistic participants scored M = 5.21 (SD = 1.2), whereas non-autistic participants scored M = 6.13 (SD = 0.7). Furthermore, two studies reported data from parents of autistic adults using the ADI-R. These studies involved the same overall sample of 1,114 autistic and 1,241 non-autistic participants; however, the scoring methods differed between the studies.

Two studies reported that the proportion of autistic adults who have at least one close friend is 60.2% and 88.3%, respectively. One study reported the presence of mutual friendships was 2% (teens) and 3% (adults). The existence of this heterogeneity may be caused by differences in information providers, as well as differences between close and mutual friends. Data from the studies using URCS suggested that the means of score was 4.67, ranging from 3.1 to 5.33. One of the comparative studies was specially reported Autistic people scored on the URCS more highly than non-autistic adults did.

In contrast to the aforementioned self-reported methods for evaluating the characteristics of friendships, Friedman et al. (52) used the ADI-R, which is based on parental reports, to evaluate friendships among autistic adults. DaWalt et al. (19) also used the ADI-R to examine quality of life within the domain of friendships. This indicated that the quality of the friendship among the autistic adults were lower than the normal population, even the other conditions (e.g., fragile X syndrome). Meanwhile, compared to the self-reported questionnaires, the data from parental reported questionnaires suggested that the number of reciprocal friends significantly less than the control group.

### Friendship practice

The pattern of preferred friendship practices and activities in autistic adults was examined in quantitative and qualitative studies. There were differences between different groups (autistic and non-autistic adults) and contexts (virtual environment). A total of 228 autistic and 232 non-autistic participants from two studies reported distinct preferences in their friendship behaviors, as evaluated using selected items from the Friendship Questionnaire (FQ). The results indicated that autistic adults generally preferred lower levels of closeness—both emotional and physical—with their friends. Furthermore, they primarily perceived their friendships as opportunities for enjoyment rather than as sources of support.

Two quantitative studies identified the primarily differences in the preferred friendship practices between the autistic and non-autistic young adults using the adopted questions from FQ, including autistic young adults would rather talk on the phone with a friend to make arrangements\meet up with a friend for a specific activity, compared to people without an ASD diagnosis prefer to talk on the phone with a friend\meet up with a friend just to chat; autistic individuals more likely to report their friends value them as someone to have fun with, compared to people without an ASD diagnosis reported their friends value them as someone to support them. Participants were most likely to visits with close friend infrequently-very few weeks (27.7%) and less than once per month (24.6%). However, they were most likely to electronic communication with close friend frequently–several times per day (29.2%).

Six qualitative studies and one mixes method study examined the friendship practices among autistic adults. Participants reported varying but generally high levels of social interest, and the difficulty and challenges in friendships as experiencing negative social situations, however, they sought and made friends in diverse ways that develop satisfying friendships. Sosnowy et al. (60) examined how autistic individuals sought to establish friendships and how they navigated through challenges and barriers. They investigated how 20 autistic adults developed satisfactory friendships with individuals who accepted and appreciated their social differences. They reported that although these individuals perceived adherence to social norms as both uncomfortable and confusing, they sought further opportunities to meet other individuals who shared their interests. They discussed the connections and potential barriers of friendships and provided examples of friendships as well as explanations of how to address the difficulties.

### Meaning of friendships

Researchers have examined the understanding and perspectives of friendships in autistic individuals through both qualitative (*n*=10) and quantitative (*n*=2) comparative research. In a quantitative study, Płatos and Pisula (12) compared gender differences in the understanding of friendships between autistic individuals and NT individuals in nonexclusive categories with six components, namely motivational (intimacy, support, and companionship) and cognitive developmental (reciprocity, unconditional responsiveness, and complexity) categories. Data from the comparative studies confirmed the existence of differences between autistic and typically developing adults in both motivational and cognitive-developmental aspects of friendship understanding, including intimacy, unconditional responsiveness, and complexity (64).

Participants described friendships in their own words across the qualitative studies, as shared interests, humor and benefits where the autism was not necessarily a central concern and the differing conceptions in excitement and motivation. Ten qualitative studies had a phenomenological or an interpretivist methodology. In two studies, interpretative phenomenological analysis (IPA; 51) were conducted, and participants reported some superordinate themes in their own words: learning the formula, socializing, challenges in friendships and bullies, and taking advantage. Gallup and Serianni (53) conducted a phenomenological study and discovered that video games provided potential support for the development of friendships and increased successful transitions.

Different concepts and meanings of friendships were explored through naturalistic observations and semi structured interviews among heterogeneous groups (including autistic individuals and NT individuals). Rossetti (56) provided descriptors of friendships and a broad conceptualization of reciprocity.

### Relationships between friendship and other factors

Six quantitative and five qualitative studies examined the relationships between friendship and other factors, including friendship as a protective factor and as an outcome. Friendship was closely related to other aspects of quality of life among autistic adults, especially loneliness and social participation. Meanwhile, the factors at individual and environmental levels also affected the status and quality of friendships.

#### Friendship as a protective factor

Multiple studies examined the positive outcomes associated with an increase in the quantity and quality of friendships, including low levels of loneliness, depression, and anxiety (20, 61); increased successful transitions and postsecondary outcomes (53); and relationship closeness (59). However, Forster and Pearson (51) and Pearson et al. (55) both focused on the interpersonal victimization, and suggested the positive and negative aspects of social relationships (e.g., friendships) among autistic adults.

#### Friendship as an outcome

At the individual level, factors such as empathy skills (41), vocabulary diversity and conversational language abilities (52), gender (45; Sedgewick et al., 2019), and autism-like traits (12, 19, 41, 45; Sedgewick et al., 2019) were examined. At the group or dyad level, additional factors were examined, including acceptance and interest sharing (60).

## Discussion

Over the last two decades, with the increasing prevalence of autism, the number of studies on autistic adults has increased. To the best of our knowledge, no systematic review has examined the experiences of friendships among autistic adults. In this scoping review, we comprehensively examined the literature to identify and summarize the characteristics of and main findings for autistic adults and to explore the gaps in the literature to guide future research. Our review included only 22 articles, indicating that the currently available evidence regarding the experiences of friendships among autistic adults is limited. In the following text, we describe our findings in detail.

First, the friendship characteristics of autistic adults were diverse and different, due to the complexity of the internal structure of friendship. Almost all quantitative studies reported that, compared with their NT peers, autistic adults reported having fewer and lower-quality friendships (41). These data were primarily obtained using the FQ (45). However, Sedgewick et al. (2019) reported autistic people scored on the URCS more highly than non-autistic adults did. Two studies used the ADI-R, which is based on parental reports. DaWalt et al. (19) reported that individuals in the FXS group were almost 12 times more likely to have a mutual friend than were individuals in the AD group. However, in qualitative research, the analysis of themes presented understanding and belonging (47). The difference in friendships between autistic and the general population was more reflected in preferred friendship practices (50), rather than just differences in scores (65).

Second, the experiences of friendships among autistic adults had different meanings, particularly in studies that involved phenomenological evidence. The experiences of friendships among autistic individuals were defined in their own words (66). Different dimensions and structures were reported in the friendship experiences of these individuals throughout their life span (25). In this scoping review, the friendships of autistic adults were examined in terms of the characteristics during adulthood that differed from those at other ages. Data obtained from different age groups were diverse [e.g., spending time with friends in social and recreational activities; (19, 41)]. The friendships among children and adolescents lied more in the exercise of social skills and participation in social life (21, 22, 24), while the friendship among autistic adults has both positive and negative sides, which is more deeply reflected in a sense of belonging and mutual understanding, or victimization.

Third, this scoping review revealed there were still some gaps in the study participants and methods on this topic and a lack of research on the context of friendships beyond the individual level. There were few research participants involving nominees (the autistic adults’ friends), and the research methods did not use data collection methods beyond language, such as Photo voice (67). In autistic adults, friendship is associated with many aspects of life. Orsmond et al. (68) reported that greater participation in social activities was predicted by characteristics of the environment, including greater maternal participation in social and recreational activities, greater number of services received, and inclusion in integrated settings while in school. This scoping review revealed that the majority of studies verified the relationship between individual characteristics, friendships, and well-being at the individual level. Only two studies reported additional factors, namely acceptance and interest sharing, at the group or dyad level (60). In our review, we discovered that few studies focused on the context in which friendships were established.

## Research gaps and future directions

Given the increase in research on the experiences of friendships among autistic adults, addressing the gaps in the literature and conducting additional research based on scoping reviews are essential.

The first gap in the literature is that the structure and characterization of autistic friendships through the voices of themselves has not been considered, which refers to their ability to freely express themselves, tell their stories, and make sense of their own life experiences (69, 70). However, the qualitative research had already involved this, the methods and tools for quantitative research still need to be enriched. There was also a lack of scales that presenting the structure of autistic friendships in measuring the friendships of autistic adults. Many of the studies included in this scoping review utilized the FQ or URCS to evaluate the status and closeness of friendships and almost all of the comparative quantitative studies compare autistic adults with the general population. As a self-report questionnaire, the FQ is based on an investigation of adults with Asperger syndrome or high-functioning autism. Generally, the FQ is appropriate for adults with average intelligence (71). Its score indicates the degree to which the respondent enjoys close, empathic, supportive, and caring friendships with other individuals (45). Using of FQ scores can mainly reflect personality differences, while using some specific questions of the questionnaire can reflect the preferential behavior of friendships. And the findings of this review were unlike the related studies in other age groups that there were similar preferences for friends and activity patterns across typical and autistic children (24). This scoping review revealed that autistic adults actually had differences in friendship preferences and the aspects in the definition of friendship compared to NT peers.

With the understanding and meaning of friendship portrayed by the experiences and worldviews of autistic adults, normative assumptions and impositions of nonautistic meanings can be deconstructed (72). Therefore, in the construction of friendship as a concept, the voice of the autistic community should be included, and the structure of this community should be examined before a questionnaire is constructed (73).

The second gap in the literature is that the related research focuses only on the context in which friendships are established. According to Sosnowy et al. (60), autistic adults tend to establish successful relationships with individuals who accept and appreciate their social differences and share their interests. The majority of studies included in this review focused on the nature of friendships among autistic adults and reported individual characteristics related to the quality of friendships, especially autism-like traits. Few studies examined the contexts (e.g., acceptance of NT peers, community, or university climate) of the friendships established by autistic adults. To achieve a more comprehensive interpretation, the context in which friendships are established should be carefully examined.

The third gap in the literature is the lack of information regarding the complex relationship between friendship and other real-life factors. According to Petrina et al. (24), multiple impairments influence the social relationships established by autistic children. Compared with NT children, children with autism tend to experience greater difficulties in developing friendships and peer relationships that are appropriate for their age. Although the majority of studies focused on the interventions used to develop friendship skills, they have not addressed the major differences between the friendships that are established during childhood and adulthood. Therefore, to examine the various patterns of friendships across an individual’s life span, additional real-life factors associated with friendships should be incorporated. In future research on the nature of friendships among autistic adults, evaluation of the various aspects of friendships can expand the concept of friendship. Further research is required to examine the complex relationship between friendships and the life experiences of autistic adults.

## Implications for policy and practice

From this scope review, it was found that there were many differences in the experiences of friendship between autistic adults and the NT population, including Less complex understanding of friendship and preferring keep physical distance and structured activities in social interactions, which were the challenges and difficulties for autistic adults to establish and maintain friendships.

Autistic adults may require autistic-led social opportunities to finding friends who accept their differences and shared interests, meanwhile, they need additional support to help them to identify and maintain the beneficial friendship, rather than the victim (74). Autistic adults may be able to establish natural relationships and networks, such as friendship, and improve their quality of life through supporting social functioning and social participation (75). Mueller et al. (76) findings highlighted the importance of externally implemented supports, including joint focus and shared interest activities and facilitated social interactions and opportunities.

In addition, there were gender and age differences in friendships experienced by autistic adults (77). Meanwhile, the apparent disparity observed between desired friendship (number of friends and time spent with friends) versus the actual friendship. It is important to consider the complexity and diversity in the internal structure of friendships experienced by autistic adults. And the finding that autistic adults scored on the URCS more highly than non-autistic adults revealed autistic adults may enjoy low-density and high-quality friendship.

## Limitations

Although this scoping review had a systematic process, it is possible that some literatures were missed. Limiting the search to literature in English excluded literature which was: published in a language other than English. It may introduce some bias, including publication and language. In addition, the included literature was focused on the concept of friendship. While some studies that used concepts related to friendship but did not examine the concept directly (e.g., social participation) might be missed, due to the complexity of friendship terms in different studies.

## Conclusion

Establishing friendships is a challenging task for autistic individuals. In this scoping review, we comprehensively examined the literature on the experiences of friendships among autistic adults. In the past decade, multiple studies examined the friendship experiences of autistic adults. By contrast, few studies compared the friendship experiences of autistic adults and children and adolescents. In this scoping review, we identified five themes of friendships among autistic adults: friendship status; friendship practice; meaning of friendships; and relationships between friendship and other factors. Although our review provides valuable insights into the friendship experiences of autistic adults, several research gaps remain to be addressed. Therefore, in the construction of friendship as a concept, the voice of the autistic community should be included, the context in which friendships are established should be examined, and the complex relationships between friendship and other real-life factors should be investigated.
